# Principal component analysis (PCA) of volatile terpene compounds dataset emitted by genetically modified sweet orange fruits and juices in which a D-limonene synthase was either up- or down-regulated vs. empty vector controls

**DOI:** 10.1016/j.dib.2016.09.003

**Published:** 2016-09-12

**Authors:** Ana Rodríguez, Josep E. Peris, Ana Redondo, Takehiko Shimada, Leandro Peña

**Affiliations:** a Departamento de Biotecnología y Mejora Vegetal de Especies Cultivadas. Instituto de Biología Molecular y Celular de Plantas - Consejo Superior de Investigaciones Científicas (IBMCP-CSIC), Av. Ingeniero Fausto Elio s/n, 46022 Valencia, Spain; b Fundo de Defesa da Citricultura, Av. Adhemar Pereira de Barros, 201, 14807–040 Vila Melhado, Araraquara, São Paulo, Brazil; c Centro de Protección Vegetal y Biotecnología. Instituto Valenciano de Investigaciones Agrarias (IVIA), carretera Moncada-Náquera Km. 4.5, 46113 Moncada Valencia Spain; d National Institute of Fruit Tree Science (NIFTS), National Agriculture and Bio-oriented Research Organization (NARO), Shizuoka 424-0292, Sizuoka, Japan

**Keywords:** PCA, Volatiles, D-limonene, Odor

## Abstract

We have categorized the dataset from content and emission of terpene volatiles of peel and juice in both Navelina and Pineapple sweet orange cultivars in which D-limonene was either up- (S), down-regulated (AS) or non-altered (EV; control) (“Impact of D-limonene synthase up- or down-regulation on sweet orange fruit and juice odor perception”(A. Rodríguez, J.E. Peris, A. Redondo, T. Shimada, E. Costell, I. Carbonell, C. Rojas, L. Peña, (2016)) [1]). Data from volatile identification and quantification by HS-SPME and GC–MS were classified by Principal Component Analysis (PCA) individually or as chemical groups. AS juice was characterized by the higher influence of the oxygen fraction, and S juice by the major influence of ethyl esters. S juices emitted less linalool compared to AS and EV juices.

**Specifications Table**

**Table:** 

Subject area	*Biology*
More specific subject area	*Genetic engineering of a terpene synthase in sweet orange alters fruit and juice odor profile and perception*
Type of data	*Figures*
How data was acquired	*Analysis by Principal Component Analysis of HS-SPME and GC–MS*
Data format	*Analyzed*
Experimental factors	Data was analyzed by PCA by using the corrected area of volatiles obtained by HS-SPME or GC–MS
Experimental features	Flavedo volatiles were captured by GC–MS while juice with pulp was analyzed by HS-SPME
Data source location	Valencia, Spain
Data accessibility	Data with this article

**Value of the data**

• Volatile identification and quantification by HS-SPME and GC–MS can be categorized by Principal Component Analysis (PCA), which is helpful in the case of analyzing different and complex profiles to map out general trends in presence, accumulation and emission of specific chemical groups [2], [3].
• We analyzed the terpene volatiles of peel and juice in both Navelina and Pineapple sweet orange cultivars with either up-, down-regulated or unaltered levels of D-limonene and related compounds. PCA can be a useful tool for rapid differentiation of fruit odors based on the comparison of volatile compound profiles [4], [5].
• The statistic aggrupation of these specific or chemical groups of volatiles is helpful in defining which ones are the most influential for odor in each transgenic line.

## 1 Data

**Fig. 1:**
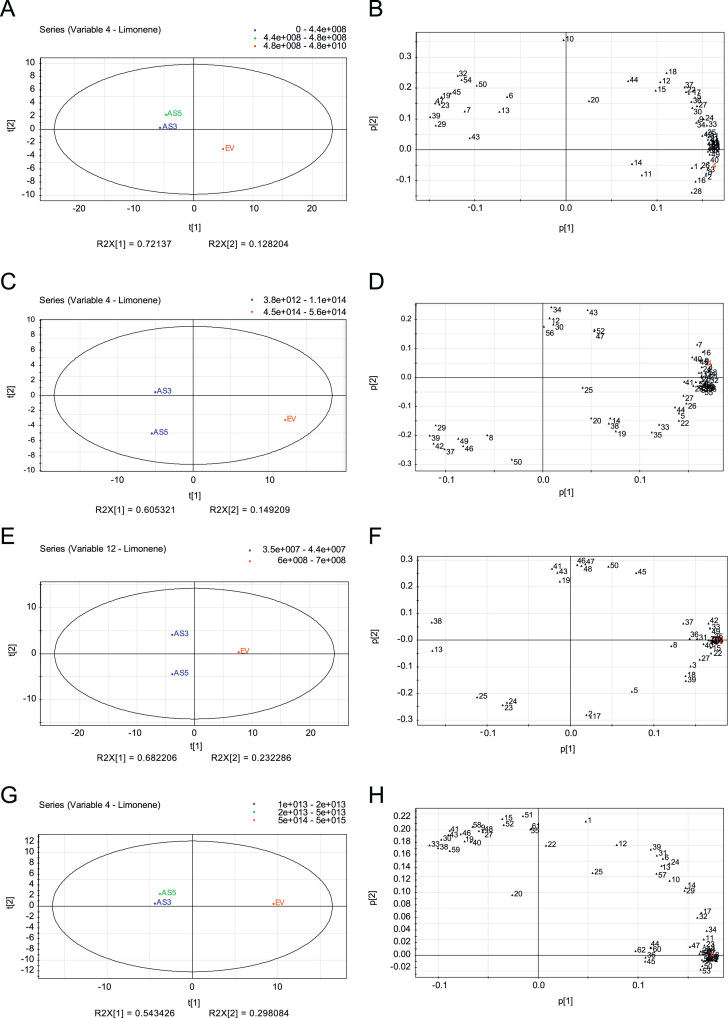
Principal Component Analysis (PCA) of volatiles from juice with pulp and flavedo of Navelina sweet orange transgenic antisense (AS3 and AS5) and empty vector (EV) lines based on chromatographic records from two seasons. (A, C, E, and G) PCA score plots (t[1] vs t[2]) of transgenic samples for the first and second principal components. (A and E) PCA score plots for the juice with pulp in the first and second season analyzed, respectively. (C and G) PCA score plots for the flavedo in the first and second season analyzed, respectively. PCA loading plots (p[1] vs p[2]) of transgenic samples for the first and second principal components. (B and F) PCA loading plots for juice with pulp in the first and second season analyzed, respectively. (D and H) PCA loading plots for flavedo in the first and second season analyzed, respectively. Each number in loading plots corresponds to a particular volatile compound, as indicated in Supplementary Table S1 and S3 of [1]. In red, the number corresponds to D-limonene.

**Fig. 2:**
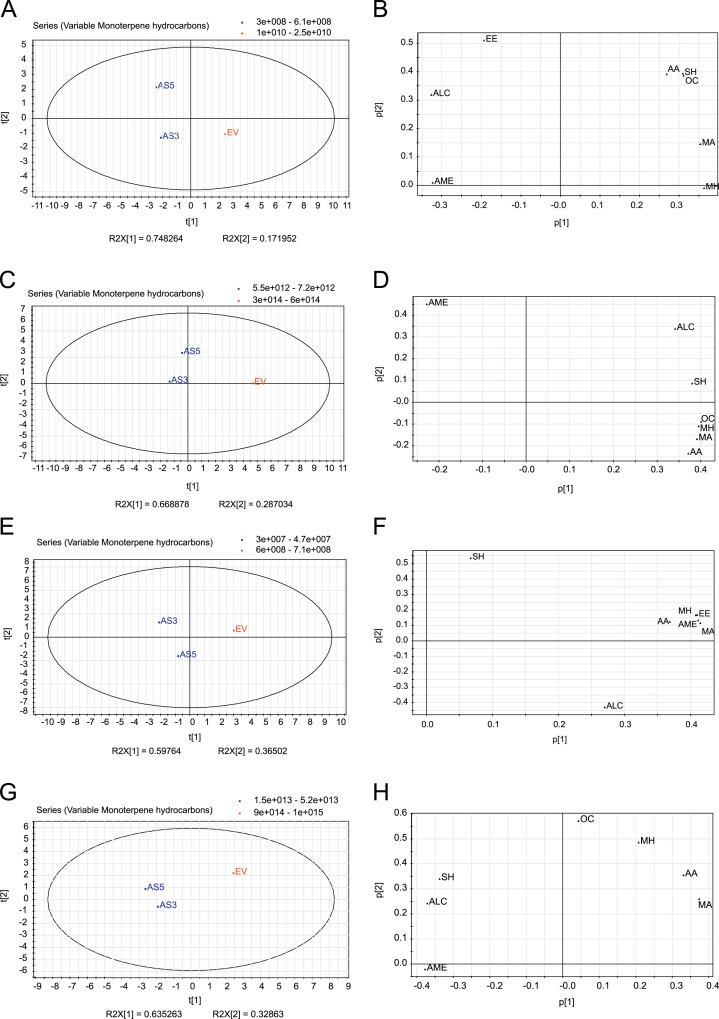
Principal Component Analysis (PCA) of chemical group of volatiles from juice with pulp and flavedo of Navelina sweet orange transgenic antisense (AS3 and AS5) and empty vector (EV) lines based on chromatographic records from two seasons. (A, C, E, and G) PCA score plots (t[1] vs t[2]) of transgenic samples for the first and second principal components. (A and E) PCA score plots for the juice with pulp in the first and second season analyzed, respectively. (C and G) PCA score plots for the flavedo in the first and second season analyzed, respectively. PCA loading plots (p[1] vs p[2]) of transgenic samples for the first and second principal components. (B and F) PCA loading plots for juice with pulp in the first and second season analyzed, respectively. (D and H) PCA loading plots for flavedo in the first and second season analyzed, respectively. Each acronym in loading plots corresponds to a particular chemical group: MH, Monoterpene Hydrocarbons; SH, Sesquiterpene Hydrocarbons; ALC: Alcohols; AA, Aliphatic Aldehydes; MA, Monoterpene Aldehydes; EE, Ethyl Esters; AME, Aliphatic and Monoterpene Esters; OC, Other Compounds as indicated in Supplementary Table S1 and S3 of [1].

**Fig. 3:**
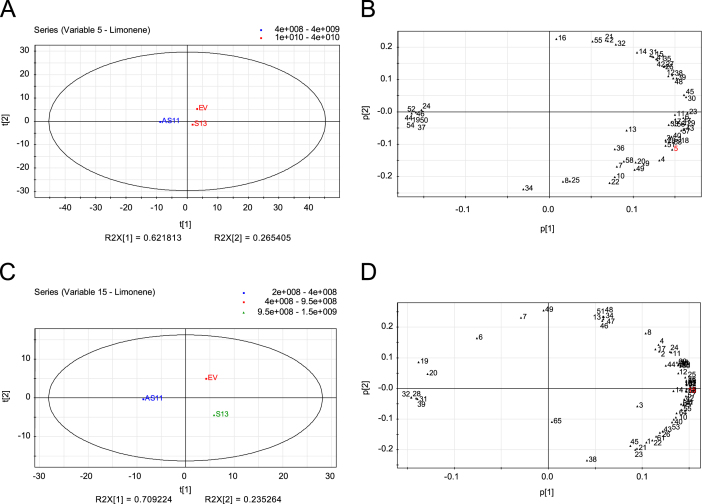
Principal Component Analysis (PCA) of volatiles from juice with pulp of Pineapple sweet orange transgenic antisense (AS11), sense (S13) and empty vector (EV) lines based on chromatographic records from two seasons. (A and C) PCA score plots (t[1] vs t[2]) for the juice with pulp of transgenic lines for the first and second principal components in the first and second season analyzed, respectively. (B and D) PCA loading plots (p[1] vs p[2]) for the juice with pulp of transgenic lines for the first and second principal components in the first and second season analyzed, respectively. Each number in loading plots corresponds to a particular volatile compound, as indicated in Supplementary Table S2 of [1]. In red, the number corresponds to D-limonene.

**Fig. 4:**
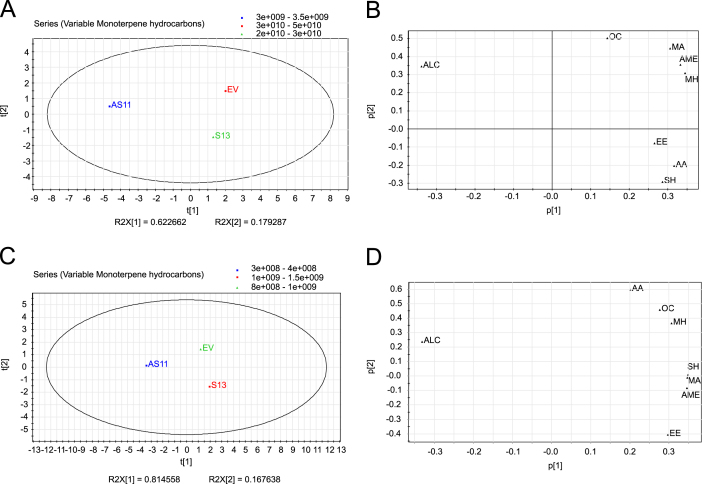
Principal Component Analysis (PCA) of chemical group of volatiles from juice with pulp of Pineapple sweet orange transgenic antisense (AS11), sense (S13) and empty vector (EV) lines based on chromatographic records from two seasons. (A and C) PCA score plots (t[1] vs t[2]) for the juice with pulp of transgenic lines for the first and second principal components in the first and second season analyzed, respectively. (B and D) PCA loading plots (p[1] vs p[2]) for the juice with pulp of transgenic lines for the first and second principal components in the first and second season analyzed, respectively. Each acronym in loading plots corresponds to a particular chemical group: MH, Monoterpene Hydrocarbons; SH, Sesquiterpene Hydrocarbons; ALC: Alcohols; AA, Aliphatic Aldehydes; MA, Monoterpene Aldehydes; EE, Ethyl Esters; AME, Aliphatic and Monoterpene Esters; OC, Other Compounds as indicated in Supplementary Table S2 of [1].

Principal component analysis (PCA) revealed two major clustering groups in Navelina flavedo and juice with pulp in both analyses from individual volatiles or from groups of compounds: the down-regulated D-limonene fruits (AS3 and AS5) vs. the non-altered control fruits (EV) (Fig. 1, Fig. 2). In Pineapple oranges, PCA showed three different clusters, the up-regulated D-limonene fruits (S), the AS fruits and the EV control fruits (Fig. 3, Fig. 4).

## 2 Experimental design, materials and methods

GC–MS and SPME-GC/MS data of the volatile content in the transgenic and control orange fruits [1] were subjected to principal component analysis (PCA) using SIMCA-P v. 11 (Umetrics, Umea, Sweden). The complete dataset of areas of volatiles including all replicates was considered. The corrected area for each compound was used for the analyses.
