# Supplementary material for: Principal component analysis (PCA) of volatile terpene compounds dataset emitted by genetically modified sweet orange fruits and juices in which a D-limonene synthase was either up- or down-regulated vs. empty vector controls
Source: Data Brief. 2016 Sep 12;9:355–61. doi: 10.1016/j.dib.2016.09.003 (PMC5031473; doi:10.1016/j.dib.2016.09.003)
Supplement: Supplementary file 1 — Supplementary material [file mmc1.docx]

Conflict of interest

None
